# Assessment of the applicability of wood anatomy and DNA barcoding to detect the timber adulterations in Sri Lanka

**DOI:** 10.1038/s41598-020-61415-2

**Published:** 2020-03-09

**Authors:** Sachithrani Kannangara, Sachinthani Karunarathne, Lahiru Ranaweera, Kalpani Ananda, Disnie Ranathunga, Hashan Jayarathne, Cholani Weebadde, Suneth Sooriyapathirana

**Affiliations:** 1 Department of Molecular Biology and Biotechnology, Faculty of Science, University of Peradeniya, 20400 Peradeniya, Sri Lanka; 2 Department of Plant, Soil and Microbial Sciences, College of Agriculture and Natural Resources, Michigan State University, East Lansing, Michigan USA

**Keywords:** PCR-based techniques, Molecular biology

## Abstract

The wood adulteration is a common problem and under-studied aspect in the timber industry of Sri Lanka. Hence we conducted a survey to assess the status of timber adulteration and check the applicability of morphometric parameters and DNA barcoding to detect the adulterated timber sources. We interviewed the stakeholders of the timber industry to collect information regarding timber adulterations. We measured the morphometric parameters; wood density and sizes of the xylem elements of the standard and adulterant species. For DNA barcoding, DNA was extracted from the wood of the selected standard and adulterant species and subjected to PCR using the markers, *matK*-*trnT* and *atpB*-*rbcL*. The PCR products were subjected to DNA sequencing. According to the survey, 92.5% of patrons, 73.7% of manufacturers and 96.7% of carpenters said timber adulteration is taking place in the country. The respondents said that the standard timber species; *Tectona grandis*, *Artocarpus heterophyllus*, and *Swietenia macrophylla*, profoundly undergo adulteration in Sri Lanka. The morphometric parameters did not discriminate the adulterant species from the standard species. The DNA barcodes *matK*-*trnT* and *atpB*-*rbcL* provided unique polymorphic DNA sequences with specific lengths for each species permitting the precise establishment of species identity and enabling the accurate detection of timber adulterations.

## Introduction

The State Timber Corporation (STC) of Sri Lanka has classified valuable timber species such as *Tectona grandis* (Teak), *Swietenia macrophylla* (Mahogany), *Artocarpus heterophyllus* (Jack) into super-luxurious and luxurious classes^1^. Because of the high demand, rareness, and prohibitions of the transportation of logs of the luxurious timber species, they are highly vulnerable for adulterations. Although the timber adulteration is a well-known issue in the country, no survey has ever conducted to document the intensity of the problem.

The most common method of timber adulteration involves substituting original timber with lower grade wood sources^2^. The common adulterant timber species are *Persea americana* (Avocado), *Mangifera indica* (Mango), *Magnolia champaca* (*Vern*. *Sapu*), *Samanea saman* (*Vern*. *Mara*) and *Toona ciliata* (*Vern*. *Toona*), and many others which are abundantly grown in Sri Lanka. The timber of the adulterant species is applied with various dyes to conjure the characteristic color patterns. The products of adulterated wood will not be able to address consumer demand. The timber adulterations result in the declination of the sales of the timber products and the violation of consumer rights. Moreover, diminishing timber sales would negatively affect the Sri Lankan economy. According to the Central Bank of Sri Lanka, wood and wood products account for the total value of USD nine million in 2013^3^. Hence authentication of timber products, which involves the accurate identification of timber species, is vital to gain the end-user trust and sustain the timber industry. However, there is no precise tool available in Sri Lanka for tracking timber adulterations.

The most commonly practiced method of timber identification is the observation of wood appearance, density, and cell anatomy. The timber inspectors use physical features such as color and grain patterns, hardness, weight, odor, luster, and texture to identify the species of a particular timber source^4^. The anatomical features such as vessel diameter, fiber length, fiber diameter, ray height, number of rays, and ray width can be measured to distinguish timber species^5,6^. However, inferences made using anatomical features are not highly accurate as morphological parameters can be varied due to the age of the tree and the environmental conditions such as water availability, nutrient content of the soil, wind, and temperature^7^. Furthermore, related timber species may contain similar morphological features, obscuring the discrimination^8^. The microscopic assessment of the timber anatomy is also time-consuming, and expensive^9,10^. The microscopic assessment of 10 timber samples requires two-days of working on replicates which costs approximately USD 275/=. The DNA barcoding of two loci of the 10 timber samples requires only a day of working which costs USD 150/= (Pers. Com).

The DNA barcoding can be used in place of physical methods for detecting timber adulterations^9,10^ serving as a rapid, accurate and automatable technique and the best way for species identification^11^. The plant DNA barcoding is done based on specific loci in the plastid, and nuclear genomes^12^. For examples, chloroplast genes *matK*-*trnT* and *atpB*-*rbcL* have been reported as the robust markers for the identification of land plants^13,14^. Because of the improvement of molecular biology and usage of automated machines that reduces the labor and the requirement of skills, DNA barcoding has become inexpensive^15^, making it more suitable for rapid identification of species. Although DNA barcoding can provide unique identity and implement in many fields in an inexpensive manner, it is not famous as a tracking method of timber adulterations. However, DNA barcoding is proven capable of detecting timber adulterations as reported for *sandalwood*^15^ and monitor illegal logging of endangered species^16^. However, the challenging step is the extraction of good quality DNA from a small amount of dried or processed wood. In many cases, the samples have to be obtained without harming the timber product. As wood is dead plant material, it only contains trace amounts of mostly degraded DNA^17^. This sort of DNA is sufficient as the template for PCR; however, the DNA quality could be low due to the presence of PCR inhibitors such as polyphenols, tannins, and resins^18,19^. However, improved methods to extract DNA from the plant tissues such as wood and seeds are currently available^20^.

In the present study, we aimed to report the status of timber adulterations in Sri Lanka using a survey, detect the typical scenarios of adulterations and reveal the inability of morphometric methods in detecting adulterations. We also targeted to demonstrate that DNA barcoding could accurately identify the timber adulterations.

## Materials and Methods

### Questionnaire guided survey

We formulated four questionnaires to collect information from patrons (i.e., customers), manufacturers, carpenters, and regulators to gather knowledge about the timber adulterations in Sri Lanka. We obtained informed consent from the participants to use the data only for the present research project. The ethical clearance for the survey was obtained from the Ethics Committee of the Postgraduate Institute of Science, University of Peradeniya, Sri Lanka. Table 1 summarizes the demographic variables and specific questions included in the four questionnaires. We collected data by interviewing randomly selected 40 patrons, 30 manufacturers/traders, 30 carpenters, and 15 regulators. The adulteration scenarios (i.e., species combinations) were selected from the survey information for morphometric and DNA analyses. All the methods employed in this study performed in compliance with the relevant guidelines and regulations imposed by the Ethics Committee of the Postgraduate Institute of Science, University of Peradeniya, Sri Lanka.

**Table 1:** Demographic information and parameters included in the four questionnaires.

Questions and variables (categorical answers in parenthesis)	P	M	C	R
**Demographic variables**				
Age in years (above 80, 80-60, 60-40, 40-20, below 20)	✓	✓	✓	✓
Gender (male, female)	✓	✓	✓	✓
Profession (government, non-government, self-employed)	✓	✘	✘	✘
Years of experience in timber trade industry (>10, 2–10, 2, less than 2)	✘	✓	✓	✓
**Timber Industry/adulteration variables**				
Interested timber product category (furniture, rafters, ceiling boards, beams, ridge plates, purling, wall plates, door/window frame, skirting, lattice [*beeralu*] and other)	✓	✘	✘	✘
Most preferred timber species (as in http://www.timco.lk/stc/)	✓	✘	✘	✘
Most dealt timber species (as in http://www.timco.lk/stc/)	✘	✓	✓	✘
Most sold timber species (as in http://www.timco.lk/stc/)	✘	✓	✓	✘
Any interest on particular timber species (completely, partially, not a concern)	✓	✓	✓	✘
Reasons for preferring specific timber species (strength and durability, luxurious look and elegance, color, trouble-free to find, uncommon, high in price/low in price)	✓	✘	✘	✘
Preference on timber (high quality original timber, low in price but with original look, only concerned about price)	✓	✓	✓	✘
Attention towards quality and standards of timber (yes, no)	✓	✓	✓	✘
Methods used to detect species/quality of timber (color and grain patterns, hardness, weight, odor, texture of grains [smooth/porous], viewing ends of wood species [end grain])	✓	✓	✓	✓
Possibility of identifying a timber species of a finished item accurately (completely, partially, impossible)	✘	✘	✘	✓
Occurrence of timber adulteration (yes - very much, yes, no) (list original and adulterant species)	✓	✓	✓	✓
Visual differences between original and adulterated timber (yes, yes - a little, no)	✓	✓	✓	✓
Adulteration methods (stain with dyes, using wood with similar grain patterns, other)	✘	✓	✓	✓
Shop owners requesting to adulterate timber products (yes, no, unknown/no experience)	✘	✘	✓	✘
Timber adulteration (it violates consumer rights therefore it has to be stopped, not a problem as they are cheap in price and easy to find, not a matter of concern)	✓	✓	✓	✓
Potential consequences of timber adulteration (less durability, less attractiveness, reduction of demand for timber)	✓	✓	✓	✓
Tracking adulterated timber (a motivation to buy timber with confidence and trust, it would be fair for consumers, it would not make any difference)	✓	✓	✓	✓

✓: question raised; ✘: not included, P: patron; M: manufacturer/trader; C: carpenter; R; regulator.

### Sample collection

We collected leaf and wood samples from eight species, *T. grandis*, *S. macrophylla*, *A. heterophyllus*, *P. americana*, *M. indica*, *M. champaca*, *S. saman*, and *T. ciliata*. The leaf samples were stored at −80 °C and wood samples at room temperature. We also photographed the representative trees, cross sections of the matured logs, twigs and leaves of all the species for the illustration purposes.

### Creation of adulteration scenarios under workshop setting

We smoothened the collected wood samples using sand papers to obtain furniture quality wood surfaces and photographed wood sections before polishing. A professional carpenter was hired to stain and polish the wood sections. A set of horizontally and vertically cut wood pieces were polished using a commercially available formula of *natural color enhancer* (i.e., top-coat or polish) without any staining for the comparison purposes. We used the commercially available *T. grandis* colorant to dye *S. saman* and *M. champaca* wood to create adulteration scenario of *T. grandis*. Furthermore, we used the commercially available colorant of *A. heterophyllus* to obtain the adulteration scenario of *A. heterophyllus* by dyeing *P. americana* and *M. indica* wood. Similarly, the adulteration scenario of *S. macrophylla* was created by coloring *P. americana*, *M. champaca*, and *T. ciliata* wood with the commercially available colorant of *S. macrophylla*. The top-coat was applied to all the stained wood surfaces. Finally, we compared the non-polished, polished, and stained and polished sections of each adulteration scenarios by taking photographs.

### Morphometric data collection

We obtained wood density measurements by cutting the wood samples into square shaped pieces and recording the length (m), height (m) and width (m) and weight (kg). We calculated the density of each wood sample using the following equation.

$${\rm{Density}}\,({\rm{kg}}\,{{\rm{m}}}^{-3})={\rm{Weight}}\,({\rm{kg}})/{\rm{Volume}}\,({{\rm{m}}}^{3})$$

Simultaneously, to compare the microscopic anatomy of wood; we obtained the xylem vessel and fiber diameter measurements. We cut very thin (10–15 µm) cross sections from each wood piece using a microtome. The sections were then kept in distilled water for five mins and transferred through a series of ethanol (25%, 50%, and 70%). Then we stained the sections with safranin for one min. The stained section was mounted on a glass slide with 50% glycerin, topped with a coverslip, and sealed with a colorless topcoat. We observed the prepared slides using a light microscope (Carl Zeiss Microscopy GmbH, SN 3150000610) and captured the microscopic images at low, medium and high powers using AxioCam ICc 5 camera and Zen lite 2.1 software (Carl Zeiss Microscopy GmbH). The xylem vessel and fiber diameters were recorded from 15 different xylem vessels and 30 different fibers using a calibrated eyepiece graticule. The vertical diameter and horizontal diameter were measured for each cell and the mean diameters were calculated for analysis.

### DNA based detection of timber adulterations

#### DNA extraction from leaves

We isolated the genomic DNA from leaves using a modified CTAB plant DNA extraction protocol according to the instructions given in Porebski *et al*.^21^ and stored the extracted DNA at −20 °C.

### DNA extraction from wood

We collected approximately 30 mg of wood dust from each timber sample into an eppendorf tube and mixed with 600 µL of extraction buffer (8 g of CTAB, 10 mL of 2 M Tris-HCl [pH 8.0], 8 mL 0.5 M EDTA, 16.36 g of NaCl and 2 g of PVP in 200 mL of extraction buffer). We incubated the tubes at 60 °C in a water bath for 30 mins with periodic mixing by gently inverting the tubes in every ten mins. After that, we added 600 µL of chloroform/isoamyl alcohol (24:1) and mixed by inverting the tubes several times. Then we centrifuged the tubes at 12,000 rpm for ten mins and transferred 500 µL of the supernatant into a sterile Eppendorf tube, and added with 300 µL of isopropanol and 50 µL of 3 M Na-acetate. The contents were mixed by inverting the tubes several times and then incubated on ice for 30 mins. We spun the mixture at 12,000 rpm for ten mins and discarded the supernatant. The DNA pellet was washed by adding 70% ethanol and inverting the tube a few times. Finally, we air dried the pellet entirely and then added 30 µL of TE + RNase (10 mg RNase in 1 mL of TE buffer). We incubated the mixture at 60 °C to dissolve DNA^22^. The quality and quantity of the extracted DNA were assessed using the absorbance ratio of 260/280 and 1% agarose gel electrophoresis.

### PCR amplification and DNA sequencing

We selected the DNA barcoding markers *matK-trnT* (5′-3′GCATAAATATAYTCCYGAAARATAAGTGG/TGGGTTGCTAACTCAATGG) and *atpB-rbcL* (5′-3′GAAGTAGTAGGATTGATTCTC/TACAGTTGTCCATGTACCAG) for the present study considering the availability of comparison sequences in public domain. The DNA samples extracted from each species were amplified using the two markers. We carried out PCR in a thermal cycler (Takara, Otsu Shiga, Japan). The PCR of 15 µL volume contained 1 × GoTaq Green Master Mix (Promega Corporation, Madison, Wisconsin, USA), 0.3 pmol of reverse and forward primers and 50 ng of template DNA. The PCR cycle consisted of the initial denaturation at 95 °C for 1.5 min, followed by 35 cycles of denaturation at 95 °C for 30 secs, annealing at 48 °C for 1 min, initial extension at 68 °C for 2 min and a final extension at 68 °C for 20 min for *matK-trnT* primers^23^ and for *atpB*-*rbcL* primer pair with initial denaturation at 94 °C for 4 min followed by 40 cycles of denaturation at 94 °C for 30 sec, annealing of primers at 45 °C for 30 sec, initial extension at 72 °C for 2 min and the final extension at 72 °C for 5 min^24^. We size-separated the amplified products in 2.5% agarose gel electrophoresis. We purified the PCR products using QIAquick PCR Purification Kit (Catalog No: 28104, Qiagen, Hilden, Germany) and cycle sequenced (3×) using the Genetic Analyzer ABI 3500 (Applied Bio Systems®).

### Data analysis

We summarized the responses gathered from patrons, manufacturers, carpenters, and regulators. We used the cross-tab procedure in the Statistical Package SPSS 16, SPSS Statistics Software, IBM ® SPSS Predictive Analytics (IBM.com) to assess the associations among variables. We calculated the percentages of respondents considering all patrons, manufacturers, carpenters, and regulators together to identify the adulterant species to the standard timber species. Based on these details, we selected the adulteration scenarios for the morphometric and DNA barcoding assessments. We subjected the wood density, xylem vessel diameter, and xylem fiber diameter data to General Linear Model (GLM) procedure in the Statistical Package SAS 9.4 (SAS Institute, Cary, NC, USA). We examined the *matk*-*trnT* and *atpB*-*rbcL* sequence data for matching lengths with bands detected in the agarose gel electrophoresis for PCR fragments generated by DNA samples extracted from leaves and wood. We aligned the forward and reverse sequences of each species using CLUSTALW alignment tool embedded in sequence alignment software MEGA 7.0^25^ to obtain the consensus sequences. We performed BLAST (https://www.ncbi.nlm.nih.gov/BLAST/) searches using the generated DNA sequences to confirm the species identity. We submitted the generated sequences to NCBI- GeneBank. During the submission process, we trimmed the sequence-edges according to the obligatory GenBank requirements (https://www.ncbi.nlm.nih.gov). Finally, we created the DNA barcodes using the barcode generating tool available at Bio-Rad (http://biorad-ads.com/DNABarcodeWeb/).

## Results

### Adulterations and relevant factors in the timber market

Table 2 displays the demographic variability of the respondents. The majority of the respondents were from the age group of 40–60 years. All patrons, manufacturers, carpenters, and regulators were mostly well experienced in their roles of the timber industry. All most all patrons looked for the species when buying timber products mainly because of the strength and durability. A total of 70.0% of the manufacturers and 86.7% of the carpenters said that coloring of low-quality timber with available dyes in local timber shops to have the look of intended luxurious timber is the most frequent form of adulteration. We got to know from 73.3% of the carpenters that the manufacturers always instructed the carpenters to adulterate timber for better profits.

**Table 2:** Summary of the demographic information and parameters included in the four questionnaires.

Variable/question	Categories	Percentage respondents			
		Patrons	Manufactures	Carpenters	Regulators
Age (years)	60–80	27.5	3.3	10.0	6.7
	40–60	52.5	40.0	53.0	66.7
	20–40	20.0	30.0	20.0	26.7
Gender	Male	77.5	80.0	100.0	100.0
	Female	22.5	20.0	0.0	0.0
Profession	Government	35.0	—	—	—
	Non-Government	32.5	—	—	—
	Self employed	7.5	—	—	—
	Unknown	25.0	—	—	—
Experience in timber trade industry	More than 10 years	—	53.3	83.3	60.0
	2–10 years	—	26.7	13.3	26.7
	2 years	—	6.7	—	6.7
	Less than 2 years	—	13.3	—	6.7
Interested categories of timber products	Furniture	27.5	—	—	—
	Multiple choices	72.5	—	—	—
Consideration of timber species in purchasing	Completely	97.5	—	—	—
	Partially	2.5	—	—	—
	Not a concern	0.0	—	—	—
Reasons for preferring specific timber species	Strength and durability	92.5	—	—	—
	Luxurious look and elegant color	32.5	—	—	—
	Trouble free to find	5.0	—	—	—
	Uncommon or uniqueness	2.5	—	—	—
Attention towards quality and standards of timber	Yes	97.5	96.7	90.0	—
	No	2.5	3.3	10.0	—
Possibility of identifying a timber species accurately	Completely	—	—	—	26.7
	Partially	—	—	—	66.7
	Impossible	—	—	—	6.7
Approaches for timber adulteration	Stain with dyes	—	70.0	86.7	—
	Using cheap wood with similar grain patterns	—	30.0	43.3	—
Do shop owners request/recommend to adulterate timber products?	Yes	—	—	73.3	—
	No	—	—	20.0	—
	Unknown	—	—	6.7	—

### Perceptions of the patrons

We identified two groups of patrons according to the purchasing interests; 72.5% of them were interested in buying multiple timber products (furniture, rafters, ceiling boards, door/window frames, and other timber-based items) and 27.5% were interested only in purchasing furniture. A total of 97.5% of the patrons paid attention to the timber species when buying the products (Table 2). Accordingly, 82.5% of the patrons mentioned *T*. *grandis* as their preferred species. The 62.5% and 40.0% of the patrons preferred *A*. *heterophyllus* and *S*. *macrophylla* respectively (Table 3). Out of the patrons, 92.5% preferred *T*. *grandis*, *A*. *heterophyllus* and *S*. *macrophylla* due to their strength and durability; whereas 97.5% indicated that quality standards of the timber products are prerequisites making the purchasing decisions (Table 2).

**Table 3:** The summary of opinions of the respondents on the concerns regarding timber adulterations in Sri Lanka.

Concerned parameter (CP)	Categories within CP	Percentage respondents			
		Patrons	Manufacturers	Carpenters	Regulators
Preferred species out of the luxurious timber species in Sri Lanka (data are shown for the three spp. selected for the study)	*T. grandis*	82.5	96.7	90.0	—
	*S. macrophylla*	40.0	50.0	83.3	—
	*A. heterophyllus*	62.5	16.7	86.7	—
Quality of the timber product looking for	High quality and original	95.0	50.0	73.3	—
	Low price and original look	10.0	46.5	13.3	—
	Only about low price	2.5	26.7	26.7	—
Parameters to rely on when checking the quality of timber product	Color and grain patterns	65.0	70.0	86.7	100.0
	Density	47.5	26.7	40.0	26.7
	Hardness	35.0	40.0	56.7	40.0
	Texture of grains	5.0	26.7	23.3	40.0
	Viewing similar grains	15.0	3.3	43.3	40.0
Awareness about timber adulteration	Yes	92.5	73.3	96.7	—
Presence of visual differences in adulterated timber	Clear visual difference	40.0	40.0	20.0	—
	No/petty visual difference	47.5	40.0	63.3	—
Opinion about timber adulteration	Violating consumer rights	90.0	70.0	73.3	86.7
	Cheap and available	7.5	20.0	23.3	6.7
	Not a matter of concern	2.5	6.7	3.3	6.7
Potential consequences of timber adulteration	Less durability	65.0	53.3	76.7	73.3
	Timber demand shrinks	17.5	23.3	16.7	40.0
	Less attractiveness	10.0	13.3	0.0	46.7
Opinion about having a reliable tracking method to detect adulterated timber species	Buoyancy and trust grows	67.5	70.0	60.0	66.7
	Timber industry uplifts	95.0	90.0	86.7	33.3

Moreover, 95.0% of the patrons looked for high-quality original timber. However, in a separate question, 10.0% of the patrons said that they might look for the products made out of cheap timber having the look of original timber (Table 3). The patrons mentioned color and grain patterns of the wood, weight-felt, and hardness as the parameters to check the timber quality. When questioned about the timber adulterations, astonishingly 92.5% of the patrons said that they know that timber adulterations are going on in the Sri Lankan timber market. However, 47.5% of the patrons said that they could not identify the timber adulterations in the finished products. In summary, timber adulteration is a serious concern according to the opinion of the patrons as 90.0% of them said it is a violation of consumer rights. Also, 95.0% of the patrons said that if an accurate method is available to detect the adulterations, it would build up the confidence and trust, and timber industry would get uplifted from its current status (Table 3).

The association analysis between the parameters assessed with respect to the opinions of the patrons revealed that the preference to purchase *T*. *grandis* products was significantly associated with the class of profession of the patrons. The choice for high-quality timber and the strength and durability of the timber product were also significantly associated. The opinion on the adulteration was significantly associated with the patrons’ concern on timber quality, awareness on the occurrence of timber adulterations and the preference for strength and durability of the timber products (P < 0.05) (Table 4).

**Table 4:** The significant associations detected among the parameters assessed in the questionnaire guided interview.

Tested association between two parameters	Calculated χ^2^ value Significant at (P ≤ 0.05)
**Patrons**	
Preference to purchase *T. grandis* products *vs*. category of profession of the respondent	13.74
Preference on high quality timber *vs*. strength and durability of the product	5.48
Opinion about timber adulteration *vs*. patron’s concern of timber quality	9.23
Opinion about timber adulteration *vs*. awareness on occurrence of timber adulteration	46.94
Opinion about timber adulteration *vs*. preference for strength and durability	16.38
**Manufacturers**	
Patron’s inquiry about timber quality *vs*. patron’s preference for *T*. *grandis*	30.00
Methods used for adulteration process *vs*. experience in timber industry	18.75
Potential consequences of timber adulteration *vs*. knowledge on occurrence of timber adulteration	11.33
Durability *vs*. proportion of timber adulteration in market	9.60
Preference of patrons *vs*. experience in timber adulteration	17.58
**Carpenters**	
Detectability of visual differences *vs*. experience in timber adulteration	8.76
Proportion of timber adulteration in market *vs*. patron’s preference on high quality timber	15.25
Potential consequences of timber adulteration *vs*. patron’s preference for *T*. *grandis*	11.11
Experience in timber trade industry *vs*. preference for the hardness of wood	11.06
**Regulators**	
Experience in years *vs*. ability to identify timber species and adulteration	18.33
Experience in years *vs*. opinion on timber adulteration	15.64
Opinion on timber adulteration *vs*. durability of timber products	6.35

### Perceptions of the manufacturers

Out of the manufacturers interviewed, 96.7% of them said that *T*. *grandis* is the most preferred and frequently used timber species. The 50.0% and 16.7% of the manufacturers also preferred *S*. *macrophylla* and *A*. *heterophyllus* respectively. The high quality of the timber and low price with the original look were essential when purchasing as revealed by 50.0% and 46.5% manufacturers respectively. Only 77.3% of the manufacturers expressed that they aware about the timber adulteration is going on in Sri Lanka and the respondents also independently agreed with the opinion of patrons stating that it is a violation of consumer rights and an accurate system for timber adulteration is necessary (Table 3).

According to the opinion of the manufacturers, the association between patrons’ inquiries about the timber quality and the preference for *T*. *grandis* was significant. The association between the experience of the manufacturers in the timber industry and methods used in adulterating procedure was also significant. The manufacturer’s perception on the preference of the patrons and the degree of experience of the manufacturers in timber industry were also significantly associated (P < 0.05) (Table 4).

### Perceptions of the carpenters

For carpenters also, *T*. *grandis* was the most popular timber species as revealed by 90.0% of them. Also, 83.3% and 86.7% of the carpenters respectively said that *S*. *macrophylla* and *A*. *heterophyllus* are popular timber species. A total of 86.7% of the carpenters said that they use color and grain patterns to check the quality of the timber and adulterants commonly used in Sri Lanka. Moreover, 63.3% of the carpenters said once adulterated; the products were finished by coloring and polishing; they cannot be differentiated from the non-adulterated products. The 73.3% of the carpenters also agreed that the timber adulterations violate the consumer rights and 86.7% said that a system is required to detect the timber adulterations to uplift the timber industry (Table 3). Interestingly, according to the opinion of the carpenters, the degree of the timber adulterations in the market and the patron preference/search for the high-quality final product were significantly associated (Table 4).

### Perceptions of the regulators

Two third of the regulators were aged between 40–60 years, and 60.0% of them had more than 10-years of experience in the timber trade industry. The regulators must recognize the type of timber during the transportation of logs, timber, and furniture or during any inspection event. All the regulators said that they use color and grain patterns for differentiation of the timber species. Moreover, 40.0% and 26.7% of the regulators employed the hardness plus texture of grains and the density respectively for the identification of the timber species. A total of 66.7% of the regulators mentioned that only partial identification accuracy is possible when inspecting adulterated timber products. Only, 26.7% of the regulators claimed that they got skills to identify the species origin of the timber samples (Table 2).

According to the opinion of regulators, the majority (86.7%) stated that timber adulteration violates consumer rights and it has to be stopped. The 73.3% of the regulators claimed that timber adulteration would lead to less durability of the product. The 46.7% and 40.0% of the regulators said that the timber adulterations would lead to less attractiveness of timber products and reduction of the demand respectively. Two third of the regulators also felt the essential need of a novel tracking method for timber identification. Totals of 66.7% and 33.3% of the regulators expressed that a novel approach of detecting timber adulteration would help to identify illegal timber and safeguard the consumer rights respectively.

Out of the parameters assessed, the experience of the regulators was significantly associated with their ability to identify adulterations and the timber species in a finished product. Also, the opinion of the regulators on timber adulteration and their perceived understanding of the durability of the timber product were significantly associated (P < 0.05) (Table 4).

### Standard and adulterant timber species

The survey respondents expressed the names of the standard species and the adulterant species according to their experience. The respondents nominated many standard and adulterated species. During the survey, the respondents had recognized eight standard high-quality timber species in Sri Lanka currently undergone adulterations. We labeled the combinations of standard species and its nominated adulterations as an adulteration scenario (AS). There is a total of eight AS identified. In AS1, *T*. *grandis* is adulterated with 12 species in which *M*. *champaca* (25.4% respondents said), *S*. *saman* (14.9%) and ten other species. Similarly, in AS2, *A*. *heterophyllus* is adulterated with *P*. *americana* (16.5%) and *M*. *indica* (13.4%) and eight more species. In AS3, *S*. *macrophylla* is adulterated with *P*. *americana* (16.4%), *M*. *champaca* (10.4%) and *T*. *ciliata* (10.4%) and five others. The full list of standard species and their adulterant species are given with the percentage respondents in Table 5.

**Table 5:** Summary of the adulterations/species identified according to the survey.

ID^$^	Standard Species with family and vernacular name in Sri Lanka	Adulterant Species with family and vernacular name in Sri Lanka	% respondents*
**AS1**	***Tectona grandis*** **L.f., Lamiaceae (*Teak*)**	***Magnolia champaca*** **L., Magnoliaceae (Sapu)**	**25.4**
		***Samanea saman*** **(Jacq.) Merr., Fabaceae (Para mara)**	**14.9**
		*Acacia planifrons* Wight & Arn., Fabaceae (Acacia)	7.5
		*Terminalia bellirica* (Gaertn.) Roxb., Combretaceae (Bulu)	6.0
		*Albizia julibrissin* Durazz., Fabaceae (Albizia)	3.0
		*Leucaena leucocephala* (Lam.) de Wit, (Ipil ipil)	3.0
		*Careya arborea* Roxb., Lecythidaceae (Kahata)	3.0
		*Vitex pinnata* L., Lamiaceae (Milla)	1.5
		*Alstonia macrophylla* Wall., Apocynaceae (Alastonia)	1.5
		*Macaranga peltata* Roxb. Mueller, Euphorbiaceae (Kenda)	1.5
		*Adina cordifolia* (Roxb.) Brandis, Rubiaceae (Kolon)	1.5
**AS2**	***Artocarpus heterophyllus*** **Lam., Moraceae (Jack)**	***Persea americana*** **Mill., Lauraceae (Avocado)**	**16.4**
		***Mangifera indica*** **L., Anacardiaceae (Mango)**	**13.4**
		*Artocarpus nobilis* Thwaites, Moraceae (Del)	12.0
		*Mangifera zeylanica* (Blume) Hook.f., Anacardiaceae (Atamba)	10.4
		*Berrya cordifolia* (Willd.) Burret, Malvaceae (Halmilla)	3.0
		*Nauclea orientalis* (L.) L., Rubiaceae (Bakme)	1.5
		*Durio zibethinus* L., Malvaceae (Duriyan)	1.5
		*Alstonia macrophylla* Wall., Apocynaceae (Alastonia)	1.5
		*Calophyllum inophyllum* L. Calophyllaceae (Domba)	1.5
		*Hevea brasiliensis* Müll.Arg., Euphorbiaceae (Rubber)	1.5
**AS3**	***Swietenia macrophylla*** **King., Meliaceae (Mahogany)**	***Persea americana*** **Mill., Lauraceae (Avocado)**	**16.4**
		***Magnolia champaca*** **L., Magnoliaceae (Sapu)**	**10.4**
		***Toona ciliata*** **M. Roem., Meliaceae (Toona)**	**10.4**
		*Pinus* sp., Pinaceae (Pinus)	1.5
		*Melia dubia* Cav., Meliaceae (Lunumedella)	1.5
		*Macaranga peltata* Roxb. Mueller, Euphorbiaceae (Kenda)	1.5
		*Cerbera odollam* Gaertn., Apocynaceae (Kaduru)	3.0
		*Albizia julibrissin* Durazz., Fabaceae (Albizia)	3.0
AS4	*Albizia odoratissima* (L.f.) Benth., Fabaceae (Sooriyamara)	*Samanea saman* (Jacq.) Merr., Fabaceae (Paramara)	4.5
		*Thespesia populnea* (L.) Sol. Malvaceae (Gansooriya)	3.0
		*Magnolia champaca* L., Magnoliaceae (Ginisapu)	3.0
AS5	*Diospyros ebenum* J.Koenig ex Retz., Ebenaceae (Ebony)	Diospyros quaesita Thwaites, Ebenaceae (Calamader)	3.0
		*Artocarpus heterophyllus* Lam., Moraceae (Jack)	1.5
		*Vitex pinnata* L., Lamiaceae (Milla)	1.5
		*Hevea brasiliensis* Müll.Arg., Euphorbiaceae (Rubber)	1.5
AS6	*Chloroxylon swietenia* (Roxb.) DC., Rutaceae (Satin)	*Alstonia macrophylla* Wall., Apocynaceae (Alastonia)	6.0
		*Adina cordifolia* (Roxb.) Brandis, Rubiaceae (Kolon)	3.0
AS7	*Diospyros quaesita* Thwaites, Ebenaceae (Calamader)	*Senna siamea* Lam., Fabaceae (Wa)	1.5
		*Albizia julibrissin* Durazz., Fabaceae (Albizia)	1.5
AS8	*Pericopsis mooniana* (Thw.) Thw., Fabaceae (Nedun)	*Melia dubia* Cav., Meliaceae (Lunumedella)	1.5
		*Alstonia macrophylla* Wall., Apocynaceae (Alastonia)	1.5
		*Albizia julibrissin* Durazz., Fabaceae (Albizia)	1.5
		*Dillenia retusa* Thunb., Dilleniaceae (Godapara)	1.5
		*Falcataria moluccana* Miq., Fabaceae (Mara)	1.5

*The percentage of respondents out of all interviewed mentioned that the given species could be a potential adulterant species to the standard species mentioned in the second column for each Adulteration Scenario (AS).

^$^ID is the assigned number to each adulteration scenario.

The AS IDs and respective standard and adulterant species given in bold case were considered for the further analysis in the present study.

Based on the quantity of usage in the timber industry of Sri Lanka, we selected of *T*. *grandis* (AS1), *A*. *heterophyllus* (AS2) and *S*. *macrophylla* (AS3) and their two major adulterants for the morphometric and DNA barcoding analyses. Because of the two matching numbers for the second adulterant species in *S*. *macrophylla* of AS3, we had to select another species for further assessment with morphometry and DNA barcoding (Table 5). The representative images of the adaxial and abaxial surfaces of the leaves, branches and fully grown trees are given in Fig. S1.

**Figure 1:**
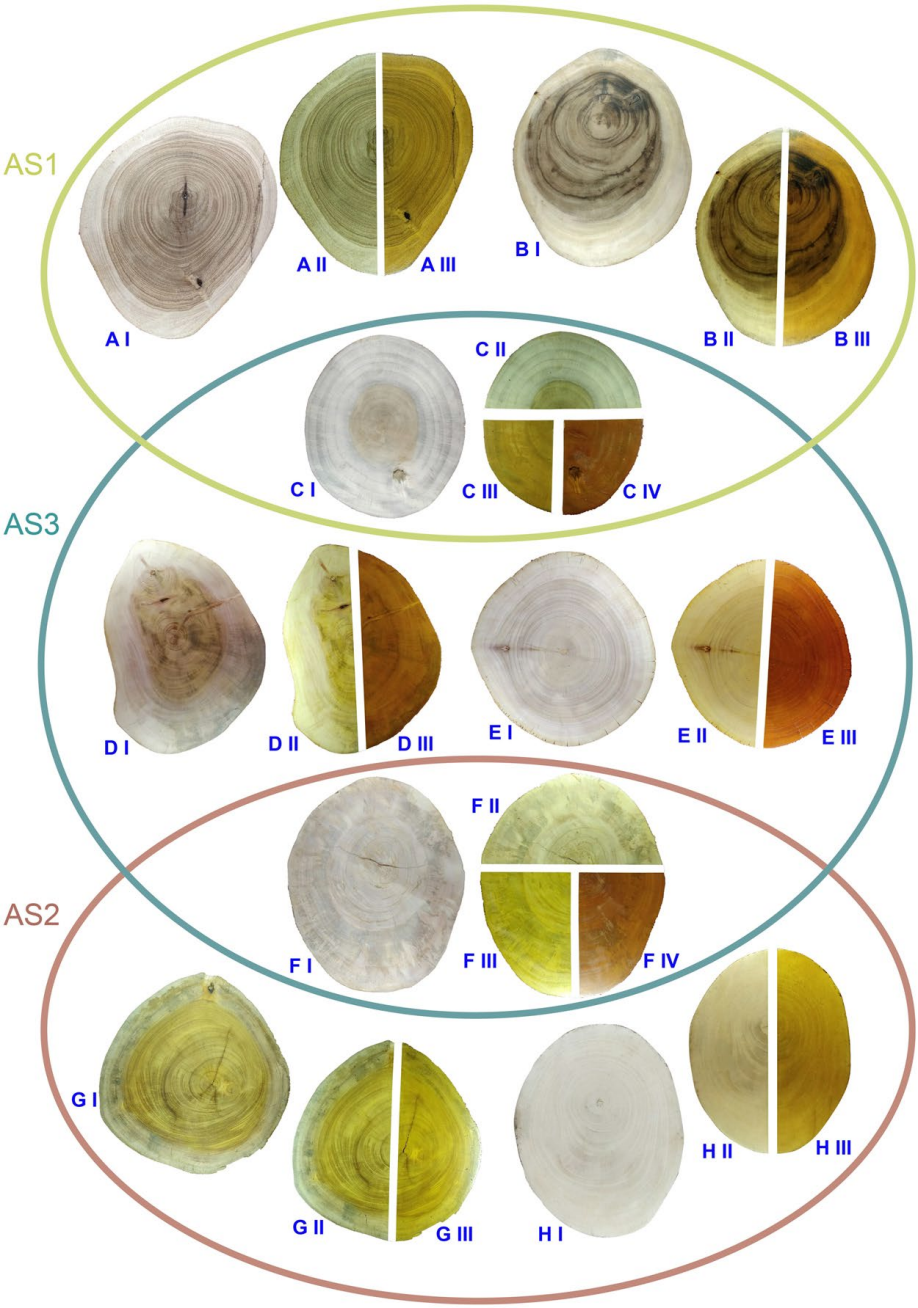
The images of cross sections of the mature logs of each species categorized into three AS (AS1, AS2, AS3). (**A**) *Tectona grandis*, (**B**) *Samanea saman*, (**C**) *Magnolia champaca*, (**D**) *Swietenia macrophylla*, (**E**) *Toona ciliata*, (**F**) *Persea americana*, (**G**) *Artocarpus heterophyllus* and (**H**) *Mangifera indica*. For each species, log sections were photographed in their original form (I), after polishing (II), after applying both dye and polish (III). In sections C and F; CIII with *T. grandis* stain, CIV with *S*. *macrophylla* stain, FIII with *A*. *heterophyllus* stain and FIV with *S*. *macrophylla* stain.

**Figure 2:**
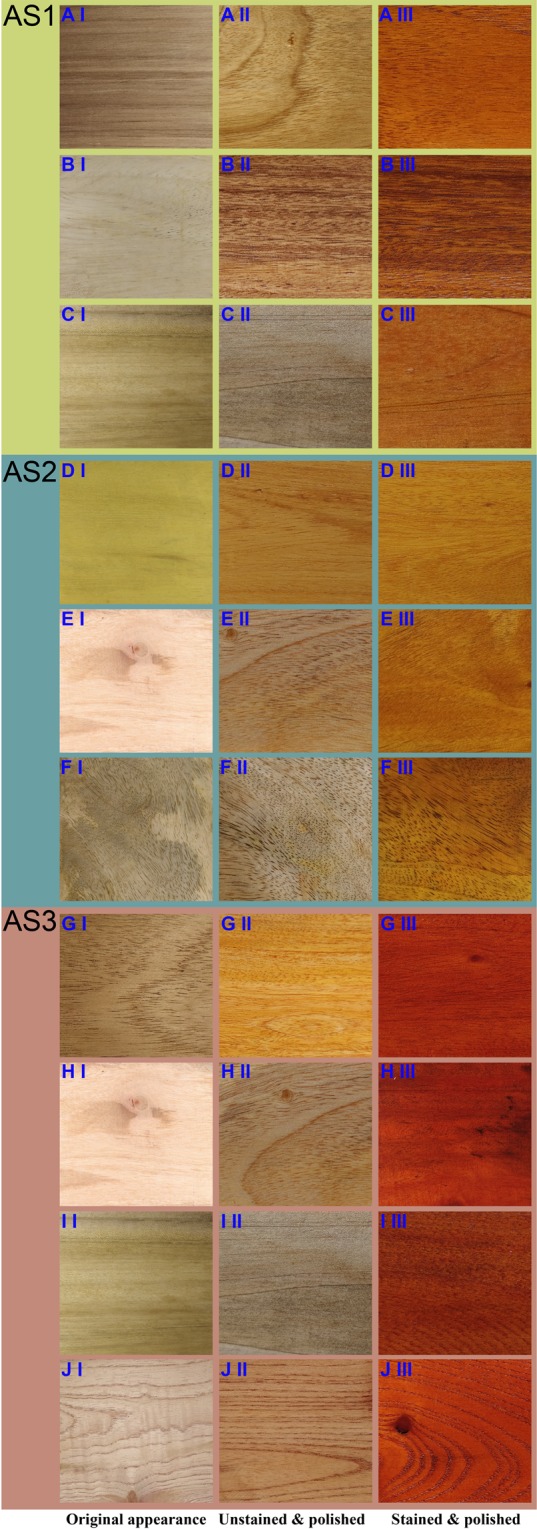
The appearance of the original and adulterated wood samples. Images of wood sections grouped into each of three AS (AS1, AS2, AS3). (**A**) *Tectona grandis*, (**B**) *Samanea saman*, (**C**) *Magnolia champaca*, (**D**) *Artocarpus heterophyllus*, (**E**) *Persea americana*, (**F**) *Mangifera indica*, (**G**) *Swietenia macrophylla*, (**H**) *P*. *americana*, (**I**) *M. champaca* and (**J**) *Toona ciliata*. For each species log sections were photographed in their original form (I), after polishing (II), after dyeing and polishing (III).

Figure 1 displays the cross sections of the matured logs with prominent secondary growth for the species selected for AS1, AS2, and AS3. A set of individual photos for the cross sections of the matured logs are given in Figs. S2–S9 for clear illustration purpose. Figure 2 summarizes the processed timber pieces (cut and smoothened with sandpapers) of the original wood of all the species, polished pieces of the three standard pieces and dyed and polished adulterant species. Accordingly, in their original forms, logs and wood sections displayed visual differences, especially in their colors. However, after application of respective dyes/stains and polishing to give them a finishing, it was evident that we could not distinguish species of the same AS by visual differences. Also, strikingly, the wood sections of *P*. *americana* and *M*. *champaca* which naturally got light colors could be successfully stained with more than one type of dyes (Figs. 1 and 2). Thus it can be shown that even using one adulterant species; two timber species could be mimicked easily. A patron with a general idea of timber would never be able to differentiate an adulterated timber.

### Detection of adulterations

#### Variation of the wood density

Table 6 shows the mean wood density of the original species and the adulterants. In AS1, although *S*. *saman* got a low mean wood density (443.67 kg/m^3^), *T*. *grandis* and *M*. *champaca* could not be discriminated as they got significantly similar mean density values. Similarly in AS2, although *P*. *americana* got the significantly least mean wood density (180.10 kg/m^3^), *A*. *heterophyllus* and *M*. *indica* were not significantly different. The same situation was observed in AS3 where *P*. *americana* got the significantly least mean wood density, however, all the other three species got significantly similar means (P < 0.05).

**Table 6:** Mean density, and diameter values of the xylem vessels and fibres.

ID	Species	Density (kg m^−3^)	Xylem vessel diameter (mm)	Xylem fiber diameter (mm)
AS1	*Tectona grandis*	653.37^a^	0.145^b^	0.006^b^
	*Magnolia champaca*	573.07^a^	0.115^b^	0.006^b^
	*Samanea saman*	443.67^b^	0.210^a^	0.011^a^
AS2	*Artocarpus heterophyllus*	680.57^a^	0.180^b^	0.011^a^
	*Persea Americana*	180.10^b^	0.106^c^	0.008^b^
	*Mangifera indica*	664.10^a^	0.277^a^	0.003^c^
AS3	*Swietenia macrophylla*	629.03^a^	0.126^a^	0.003^d^
	*Persea Americana*	180.10^b^	0.106^b^	0.008^b^
	*Magnolia champaca*	573.07^a^	0.115^b^	0.006^c^
	*Toona ciliate*	605.90^a^	0.155^a^	0.017^a^

Means denoted by same letters within column variable and within AS are not significantly different at P < 0.05.

#### Variation of the xylem vessel and fiber diameters

Table 6 also shows the mean xylem vessel diameter (XVD) of the original species and the adulterants. In AS1, although *S*. *saman* got significantly higher XVD (0.210 mm), *T*. *grandis* and *M*. *champaca* could not be discriminated as they got significantly similar mean XVD values. In AS2, *P*. *americana* got the significantly least mean XVD (0.106 mm) while *A*. *heterophyllus* got the mean XVD of 0.180 mm which was significantly less than the XVD of *M*. *indica* (0.277 mm). The same situation was observed in AS3 where *P*. *americana* got the significantly least mean wood density, however, all the other three species got significantly similar mean XVDs (P < 0.05). In AS3, the mean XVDs of *S*. *macrophylla* and *T. ciliata* were significantly similar and collectively higher than those of *P*. *americana* and *M*. *champaca* (P < 0.05).

Moreover, Table 6 depicts xylem fiber diameter (XFD) measurements which support the failure to distinguish species in AS1 as suggested by XVD measurements. Accordingly, *T*. *grandis* and *M*. *champaca* could not be distinguished from one another as they possess the same mean XFD value of 0.006 mm. The vessels of *T. grandis* and *M. champaca* were arranged in a solitary manner, while vessels of *A. heterophyllus*, *P. americana*, *M. indica* and *S. macrophylla* were partly solitary, where both single and multiple vessels were present. *T. ciliata* wood contained deviated microscopic characters from above two categories where vessels were present solitarily in low numbers and contained a high amount of fibers. Contrastingly, *S. saman* had a special arrangement where vessels were clustered together (Fig. S10).

#### Polymorphism of PCR bands and DNA barcodes

**Figure 3:**
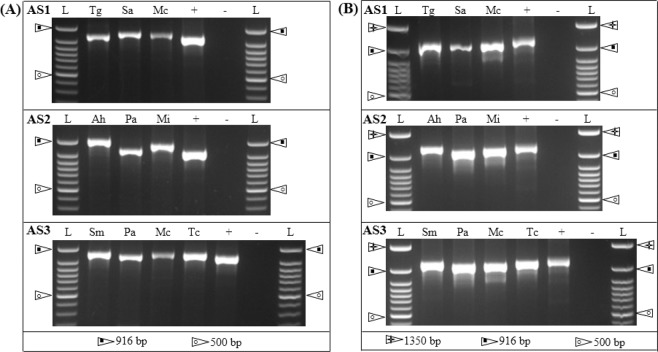
The length polymorphism of DNA barcoding loci obtained using template DNA extracted from wood samples (size separated in 2.5% agarose gel electrophoresis). (**A**) *matK-trnT*; (**B**) *atpB-rbcL*. AS1 (Tg: *Tectona grandis*, Sa: *Samanea saman*, Mc: *Magnolia champaca*); AS2 (Ah: *Artocarpus heterophyllus*, Pa: *Persea americana*, Mi: *Mangifera indica*; AS3 (Sm: *Swietenia macrophylla*, Pa: *P*. *americana*, Mc: *M. champaca* and Tc: *Toona ciliata*). L: 50 bp ladder; +: rice leaf DNA as positive control; −: negative control (i.e. without template DNA). Two/three of the standard band sizes of the ladder are shown two/three types of symbolic arrowheads.

Figures 3A and S11 depict the length polymorphism of the *matK-trnT* locus for leaf and wood DNA arranged into three adulteration scenarios (AS1, AS2, and AS3) respectively. In AS1, *T*. *grandis* had a band of ~800 bp in length while its adulterants *S*. *saman* and *M*. *champaca* had bands of ~850 bp in length. In AS2, *A. heterophyllus* provided a band of ~925 bp in length which ran behind the two bands provided by its adulterants *P*. *americana* (~820 bp) and *M*. *indica* (~890 bp). *S*. *macrophylla* of AS3 amplified a region of ~900 bp in length whose band was longer than that of its adulterants *P. americana, M. champaca* and *T. ciliata* (~890 bp).

Figures 3B and S12 show the length polymorphism of the *atpB-rbcL* locus for leaf and wood DNA categorized into AS1, AS2, and AS3 respectively. Similar to that of *matK-trnT*, we detected length polymorphisms in *atpB-rbcL* in all three adulteration scenario classes revealing the applicability of using *atpB-rbcL* locus to differentiate adulterants from their original timber species. Accordingly, in AS1, *S*. *saman* and *M*. *champaca* had shorter bands of ~930 bp in length compared to that of *T*. *grandis* (~1000 bp). In AS2, the longest-amplified band of ~1050 bp corresponded to *A. heterophyllus* while its two adulterants *P*. *americana* and *M*. *indica* provided bands of ~920 bp and ~950 bp respectively. In AS3, *S*. *macrophylla* amplified a region of ~950 bp in length whose band was longer than those of its adulterants *P. americana, M. champaca* and shorter than that of *T. ciliata* (~960 bp) (Figs. 3, Figs.S11 and S12). The DNA barcodes also provided a distinct species specific variation with length polymorphism enabling the use of DNA based detection methodology as the routine platform for detection of timber adulterants (Fig.S13). The generated DNA sequences were deposited in GenBank under the Accession Numbers MK264363-MK264378.

## Discussion

The present study reports the status of timber adulteration in Sri Lanka and the applicability of DNA barcoding using the DNA extracted from timber samples to detect the frequent adulterants. The DNA barcoding has been identified as a promising tool to detect timber adulterants^26–29^. Dev *et al*.^15^ has employed DNA barcoding to authenticate *Santalum album* L. (East Indian *sandalwood*) samples in the States Karnataka, Kerala and Tamil Nadu of India. However, the present study is the first comprehensive report that provides survey information to identify the adulterant species of wood. We focused on the three luxurious timber species; *T. grandis*, *A. heterophyllus*, and *S. macrophylla* because they are the most popular timber sources (Table 3) and highly prone to timber adulteration in Sri Lanka (Table 5)^1^. The four stakeholders; patrons, manufacturers, carpenters, and regulators have explicitly mentioned the need for an effective method to authenticate the use of luxurious/standard species in furniture/timber industry. Currently, manufacturers and carpenters employ staining and polishing techniques to conjure the characteristic appearances of the luxurious wood (Table 2)^15^. The differentiation of original timber from the adulterated counterparts is ambiguous. The patrons, carpenters, manufacturers, and regulators collectively stated that they employ visual features such as color and grain patterns, weight, hardness, texture of the end grains in cut-edges and appearance of the end grains for timber differentiation (Table 3). Moreover, most of the stakeholders believed that there are distinct visual differences between original and adulterated timber. Thus, to assess this opinion, we created adulteration scenarios in the laboratory. However, we found that once adulterated; timber species cannot be differentiated from one another (Figs. 1, 2 and Figs. S2–S9) implying the necessity of a standard protocol to detect timber adulterations.

The conventional parameters; wood density, xylem vessel diameter, and xylem fiber diameter are not reliable in detecting timber adulterations (Table 6). However, we observed significantly lower wood density for *S. saman* (443.67 kg m^−3^) than those of the other two species in AS1. In AS2 and AS3, we identified the wood density of *P. americana* was very low (180.10 kg m^−3^) compared to original species; *A. heterophyllus* and *S. macrophylla* wood (Table 6). The wood density of a tree species depends on both specific gravity and moisture content^30^. The xylem vessel diameter can only differentiate timber species in AS2 from one another while mean xylem fiber diameter can distinguish members of both AS2 and AS3. However, like density, the xylem element dimensions also could be varied based on the environmental factors and the age of the tree. Therefore, morphometric methods of species identification are not suitable for detecting timber adulterations mainly due to the lack of precision and the difficulty in obtaining sufficient samples for detection especially from the furniture^31^.

The DNA barcoding is the most established molecular technique for rapid and accurate identification of species in modern biological applications^11^ such as cataloging endangered and cryptic species, metagenomic studies, controlling pests, tracking invasive species and assessing food quality^32–34^. In the timber industry, the DNA barcoding has been used to identify the species which are threatened by illegal logging^10,16,35^. Thus, the possibility of employing DNA barcoding to detect timber adulterants reported in the present study would make a dramatic change in the future timber industry of Sri Lanka.

In the present study, we evaluated three luxurious timber species for the utility of the *matK-trnT* and *atpB*-*rbcL* barcoding markers as molecular tools for detecting timber adulterations. We selected *matK-trnT* and *atpB*-*rbcL* due to their high universality, amplification and sequencing success rates^23,24^. The banding profiles of each adulteration scenario for both leaf and wood DNA show the length polymorphisms for both loci among all timber species (Fig. 3, Figs. S11 and S12). DNA sequencing results confirmed the band lengths observed in the gels. Therefore, in cases where fast detection of adulterant species is required, PCR and gel electrophoresis can be used without DNA sequencing for the species tested in the present study.

**Figure 4:**
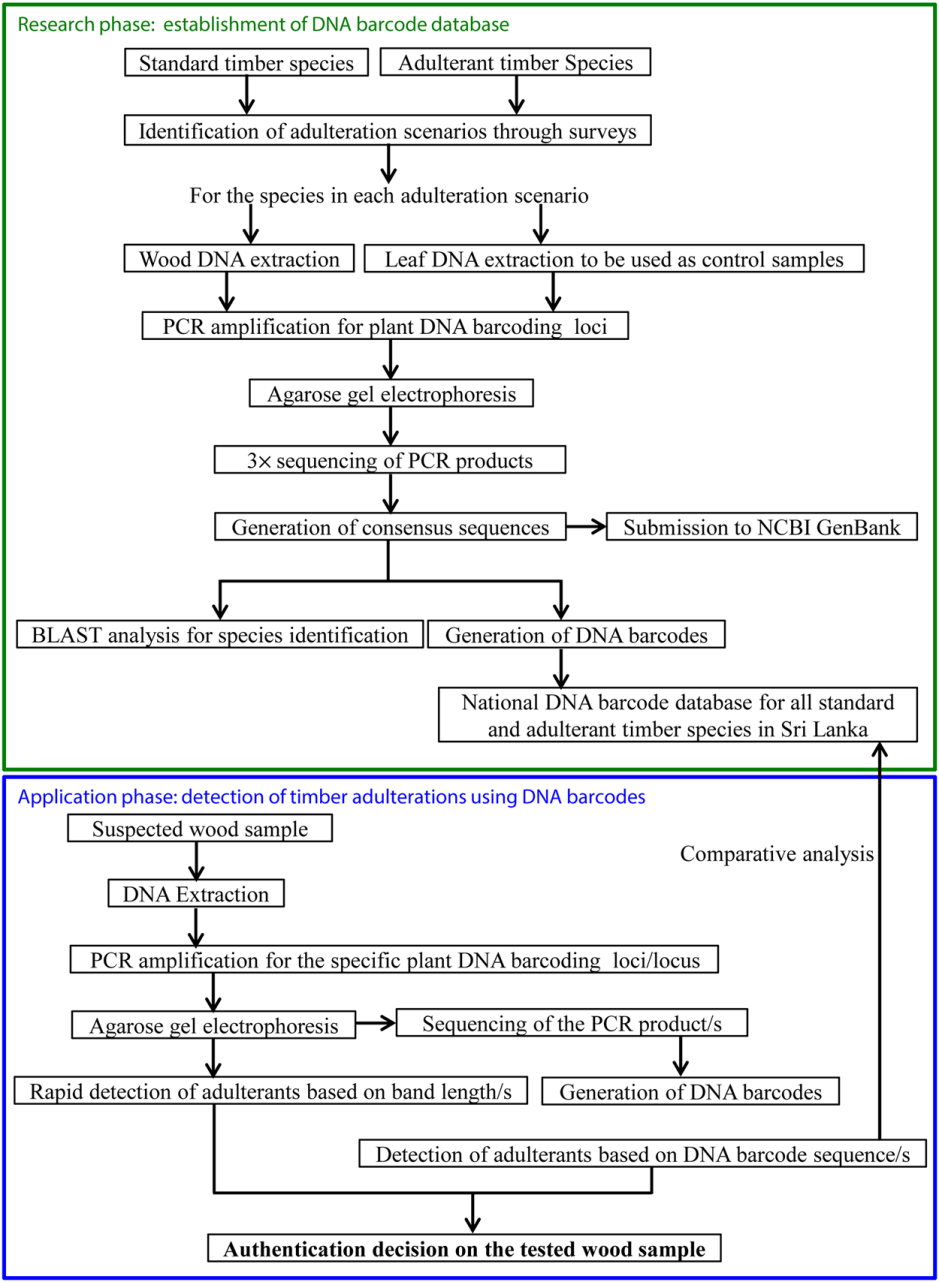
Proposed strategy involving basic research and routine application to detect timber adulteration in Sri Lanka using DNA barcoding.

Based on the experience of the present study, we propose a strategy to control and detect the timber adulterations. The proposed strategy consists of research and application phases. Initially, basic research has to be undertaken to identify all the adulteration scenarios and detect the polymorphic DNA barcodes. The generated DNA barcodes must be organized into a national database for all the timber species. In the application phase, when regulators or consumer protection agencies come across with a suspected wood sample, they can get the service from a DNA laboratory to get the relevant DNA barcode reads and search them across the established national database for matches. If the length polymorphisms exist for the PCR bands, the rapid detection of species is also possible when immediate DNA sequencing facility is not available. The regulators can provide the decision on the species identity/adulterations to the judiciary systems with 100% accuracy to prosecute the racketeers (Fig. 4).

## Conclusions

The timber adulteration is a frequent problem in Sri Lankan timber industry as revealed by 92.5% of patrons, 73.7% of manufacturers and 96.7% of carpenters interviewed in the present study. The survey also revealed that the three luxurious timber species; *T. grandis*, *A. heterophyllus* and *S. macrophylla* are highly subjected to timber adulteration than the other standard timber species in Sri Lanka. During the survey, 25.4% and 14.9% of respondents stated that *M*. *champaca* and *S*. *saman* as the major adulterants of *T*. *grandis* respectively; 16.4% and 13.4% of respondents stated *P*. *americana* and *M*. *indica* as the major adulterants of *A*. *heterophyllus* respectively. The 16.4% respondents stated that *P*. *americana* is the major adulterant species of *S. macrophylla*, whereas, 10.4% each stated *M*. *champaca* or *T*. *ciliata* as the second major adulterants. The mean xylem vessel diameters only differentiate adulterants of *A. heterophyllus* while xylem fiber diameters differentiate adulterants of both *A*. *heterophyllus* and *S*. *macrophylla*. However, xylem fiber diameters failed to distinguish *T*. *grandis* from its adulterants. The *matK-trnT* and *atpB*-*rbcL* barcodes generated polymorphic DNA sequences with specific lengths for each species enabling the precise establishment of species/timber identity using DNA barcoding.

## Supplementary information

Supplementary information.

## Data Availability

The generated sequences were submitted to GenBank under the Accession Numbers MK264363-MK264378. The other numeric and image data are available with the corresponding author (SS).
