# Supplementary material for: Assessment of the applicability of wood anatomy and DNA barcoding to detect the timber adulterations in Sri Lanka
Source: Sci Rep. 2020 Mar 9;10:4352. doi: 10.1038/s41598-020-61415-2 (PMC7062781; doi:10.1038/s41598-020-61415-2)
Supplement: Supplementary file 1 — Supplementary information. [file 41598_2020_61415_MOESM1_ESM.pdf]

Assessment of the applicability of wood anatomy and DNA barcoding to detect the timber adulterations in Sri Lanka

Sachithrani Kannangara[1], Sachinthani Karunarathne[1], Lahiru Ranaweera[1], Kalpani Ananda[1], Disnie Ranathunga[1], Hashan Jayarathne[1], Cholani Weebadde[2], Suneth Sooriyapathirana[1*]

[1]Department of Molecular Biology and Biotechnology, Faculty of Science, University of Peradeniya, 20400, Peradeniya, Sri Lanka

[2]Department of Plant, Soil and Microbial Sciences, College of Agriculture and Natural Resources, Michigan State University, East Lansing, Michigan, USA

*Corresponding author
sunethuop@gmail.com (SS)
ORCID 0000-0002-5592-1742

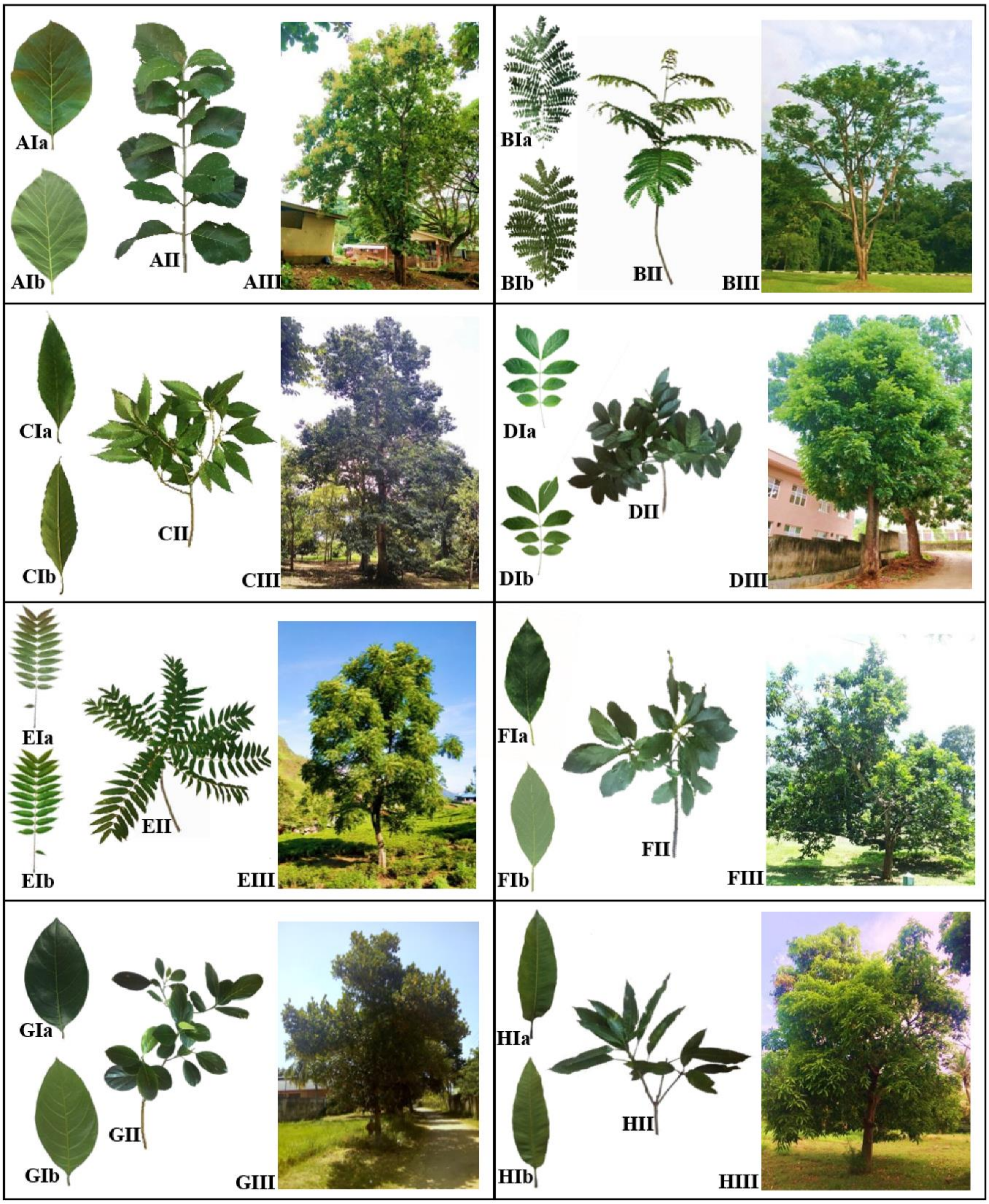

S1 Fig. The luxurious and adulterant timber species assessed in the study. A: *Tectona grandis*, B: *Samanea saman*, C: *Magnolia champaca*, D: *Swietenia macrophylla*, E: *Toona ciliata*, F: *Persea americana*, G: *Artocarpus heterophyllus* and H: *Mangifera indica*. I: leaf morphology with adaxial (a) and abaxial (b) views, II: a twig of a branch showing the leaf arrangement and III: an image of selected fully grown tree. Images are not according to a common scale.

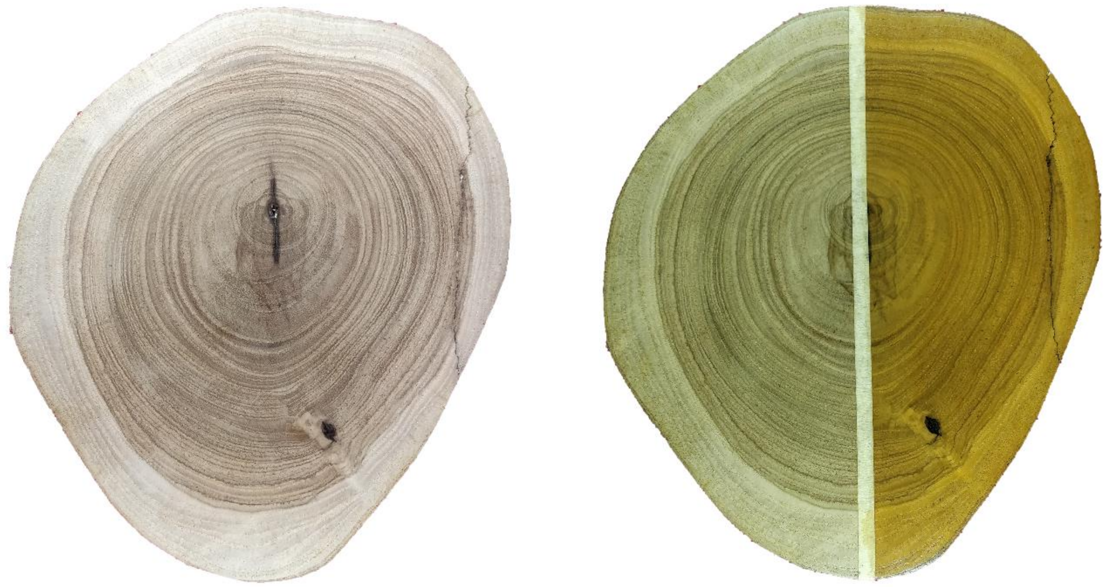

S2 Fig. A cross section of a log of *Tectona grandis*. Original appearance (Image at left); Unstained and polished (left half of the image at right); Stained and polished (right half of the image at right).

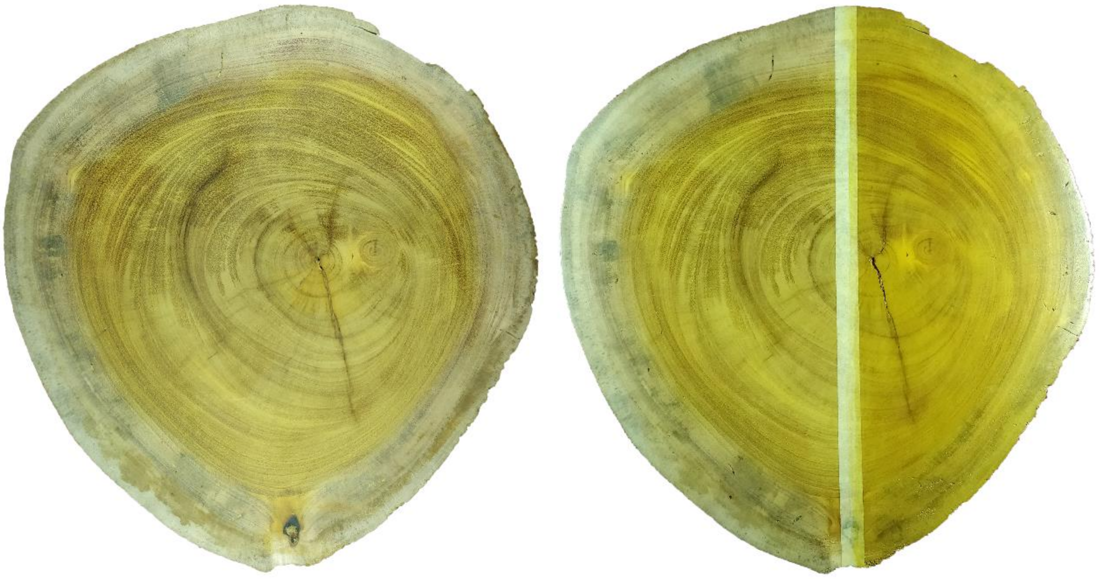

S3 Fig. A cross section of a log of *Artocarpus heterophyllus*. Original appearance (Image at left); Unstained and polished (left half of the image at right); Stained and polished (right half of the image at right).

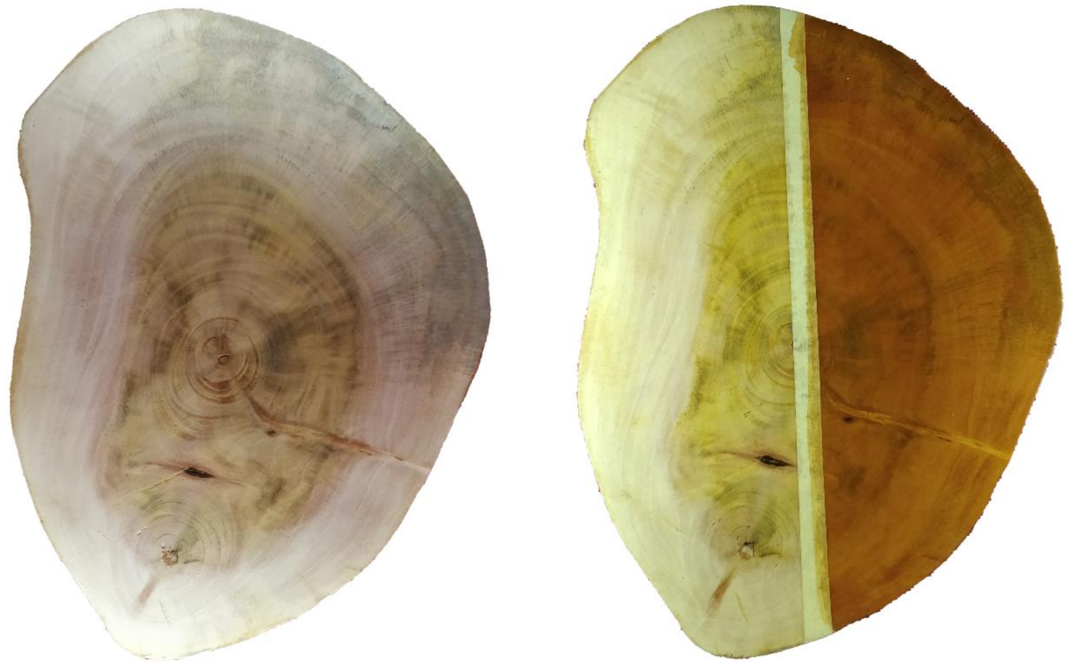

S4 Fig. A cross section of a log of *Swietenia macrophylla*. Original appearance (Image at left); Unstained and polished (left half of the image at right); Stained and polished (right half of the image at right).

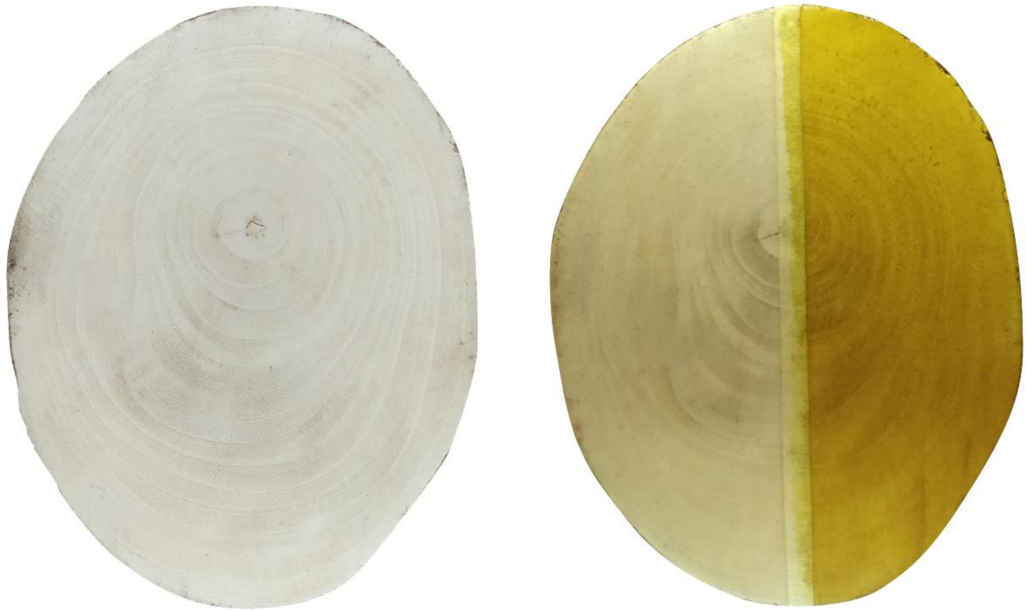

S5 Fig. A cross section of a log of *Mangifera indica*. Original appearance (Image at left); Unstained and polished (left half of the image at right); Stained and polished to adulterate the wood of *Artocarpus heterophyllus* (right half of the image at right).

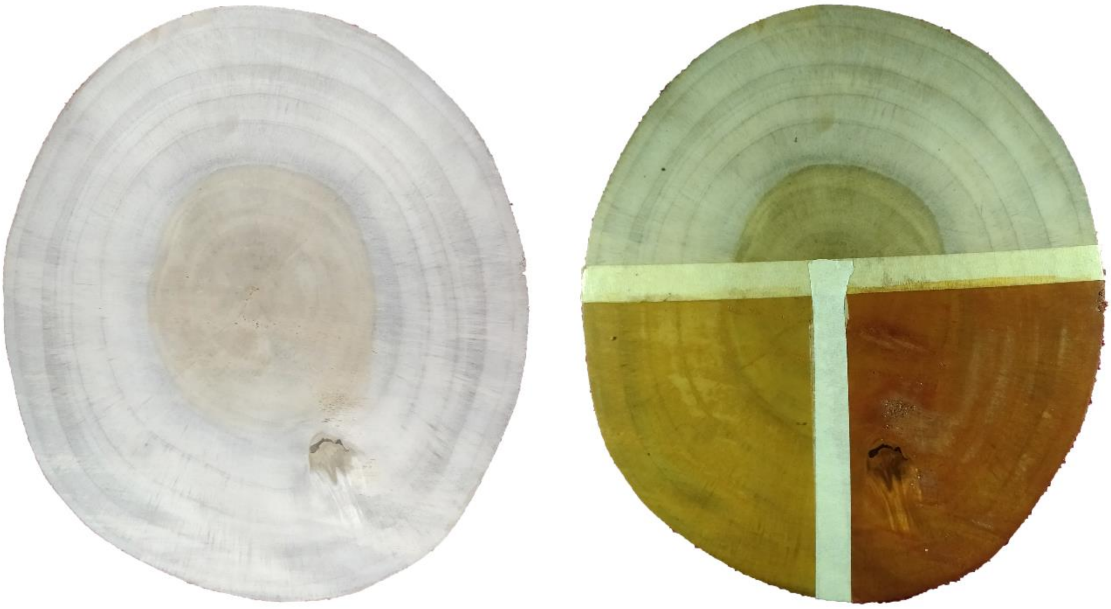

S6 Fig. A cross section of a log of *Magnolia champaca*. Original appearance (Image at left); Unstained and polished (top half of the image at right); Stained and polished to adulterate the wood of *Tectona grandis* (bottom left quarter of the image at right); Stained and polished to adulterate the wood of *Swietenia macrophylla* (bottom right quarter of the image at right).

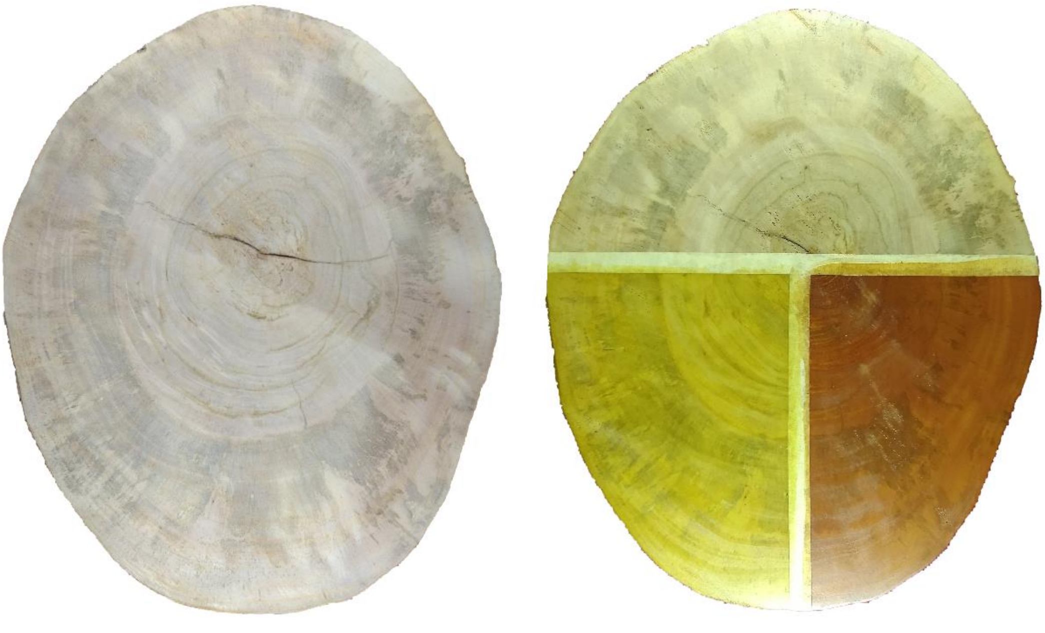

S7 Fig. A cross section of a log of *Persea americana*. Original appearance (Image at left); Unstained and polished (top half of the image at right); Stained and polished to adulterate the wood of *Artocarpus heterophyllus* (bottom left quarter of the image at right); Stained and polished to adulterate the wood of *Swietenia macrophylla* (bottom right quarter of the image at right).

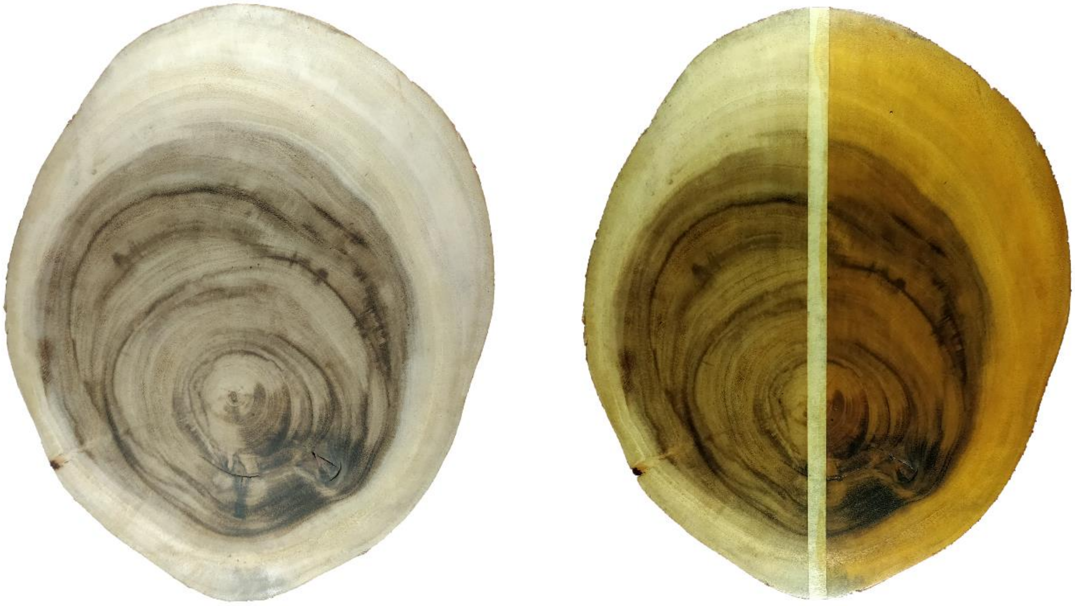

S8 Fig. A cross section of a log of *Samanea saman*. Original appearance (Image at left); Unstained and polished (left half of the image at right); Stained and polished to adulterate the wood of *Tectona grandis* (right half of the image at right).

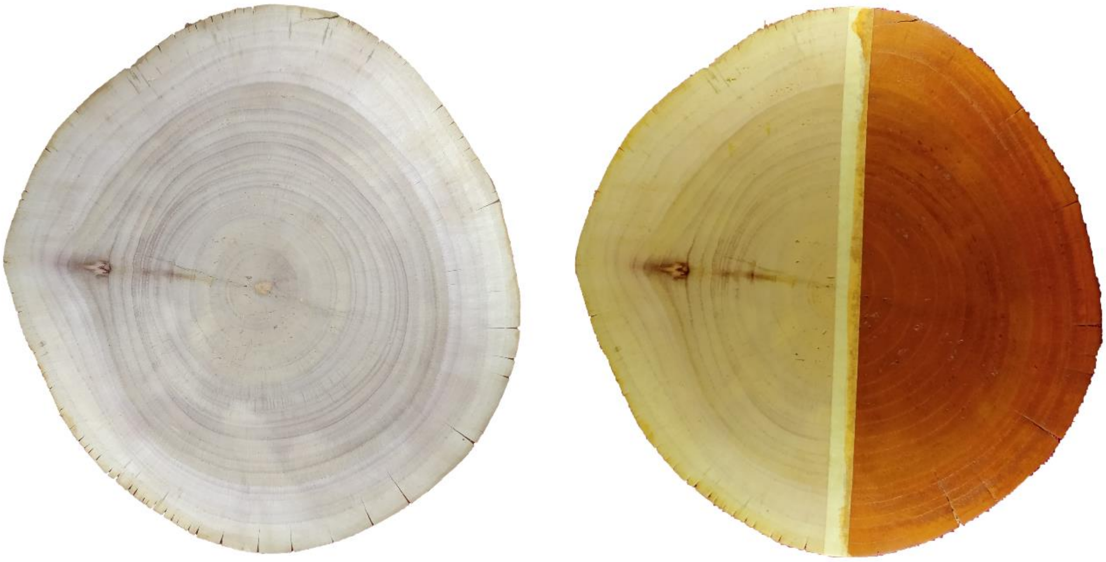

S9 Fig. A cross section of a log of *Toona ciliata*. Original appearance (Image at left); Unstained and polished (left half of the image at right); Stained and polished to adulterate the wood of *Swietenia macrophylla* (right half of the image at right).

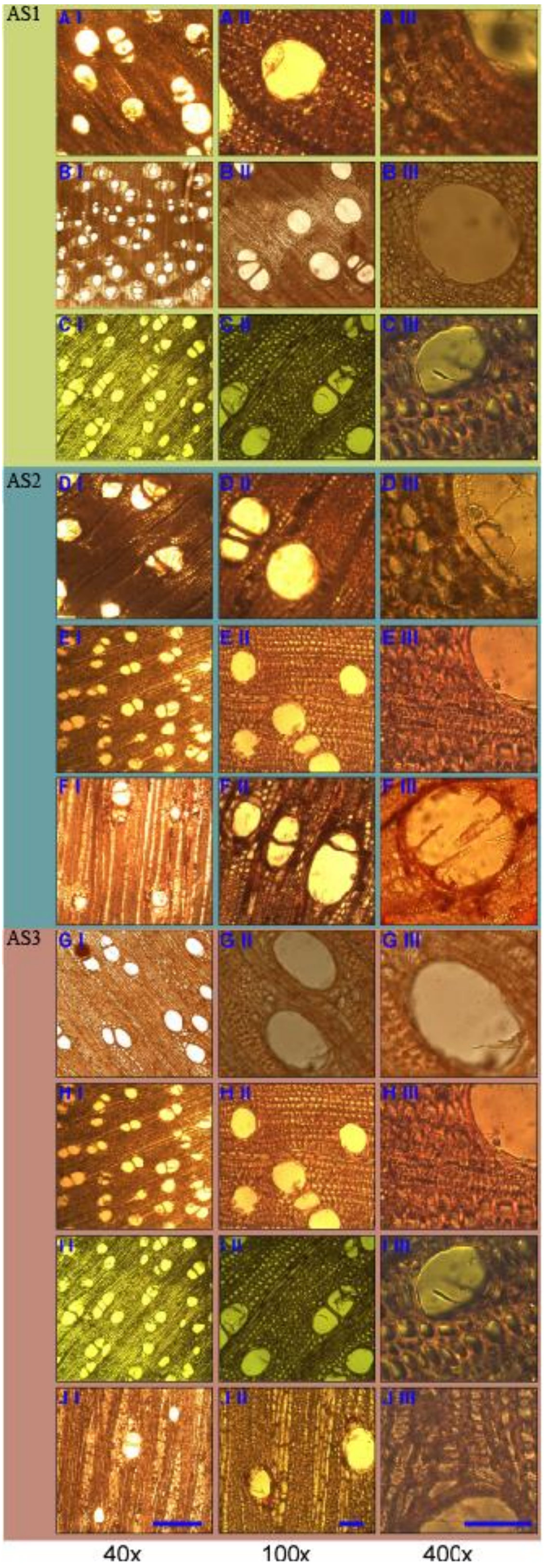

S10 Fig. Microscopic images of wood sections of each AS (AS1, AS2, AS3) obtained from thin wood sections using a microtome. A: *Tectona grandis*, B: *Samanea saman*, C: *Magnolia champaca*, D: *Artocarpus heterophyllus*, E: *Persea americana*, F: *Mangifera indica*, G: *Swietenia macrophylla*, H: *P. americana*, I: *M. champaca* and J: *Toona ciliata*. Each section was 10-15 µm thin, stained with safranin, mounted on clean glass slides with 50 % glycerin and observed under the power ×40 (I), × 100 (II) ×400 (III) of the light microscope. Scale bars are shown in the bottom of the image (×40;100 µm, ×40:10 µm, ×400:10 µm).

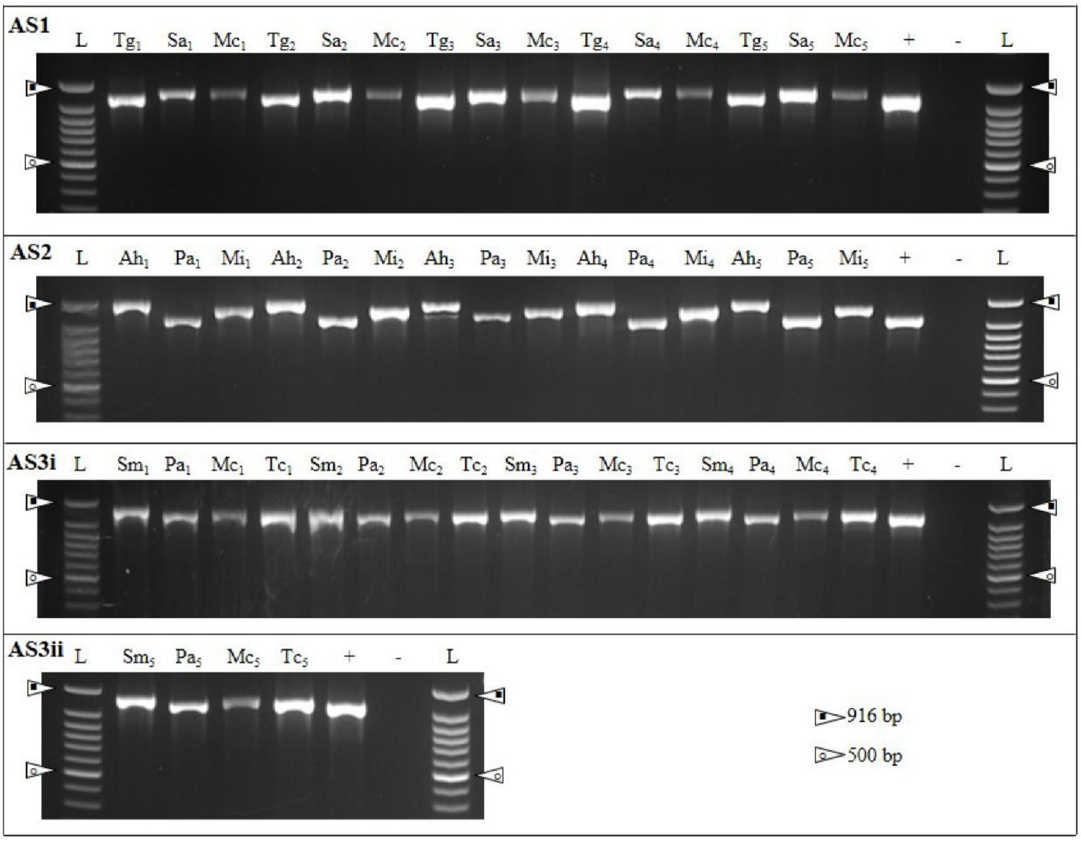

S11 Fig. ***matK-trnT*** **length polymorphism (size separated in 2.5 % agarose gel electrophoresis) obtained using template DNA extracted from immature leaves.** AS1 (Tg: *Tectona grandis*, Sa: *Samanea saman*, Mc: *Magnolia champaca*); AS2 (Ah: *Artocarpus heterophyllus*, Pa: *Persea americana*, Mi: *Mangifera indica*; AS3 (Sm: *Swietenia macrophylla*, Pa: *P. americana*, Mc: *M. champaca* and Tc: *Toona ciliata*). Five trees from each species (1→5 in subscript numbers) are shown. L: 50 bp ladder; +: rice DNA as template DNA for positive control; -: negative control (i.e. without template DNA). Two of the standard band sizes of the ladder are shown two types of symbolic arrowheads.

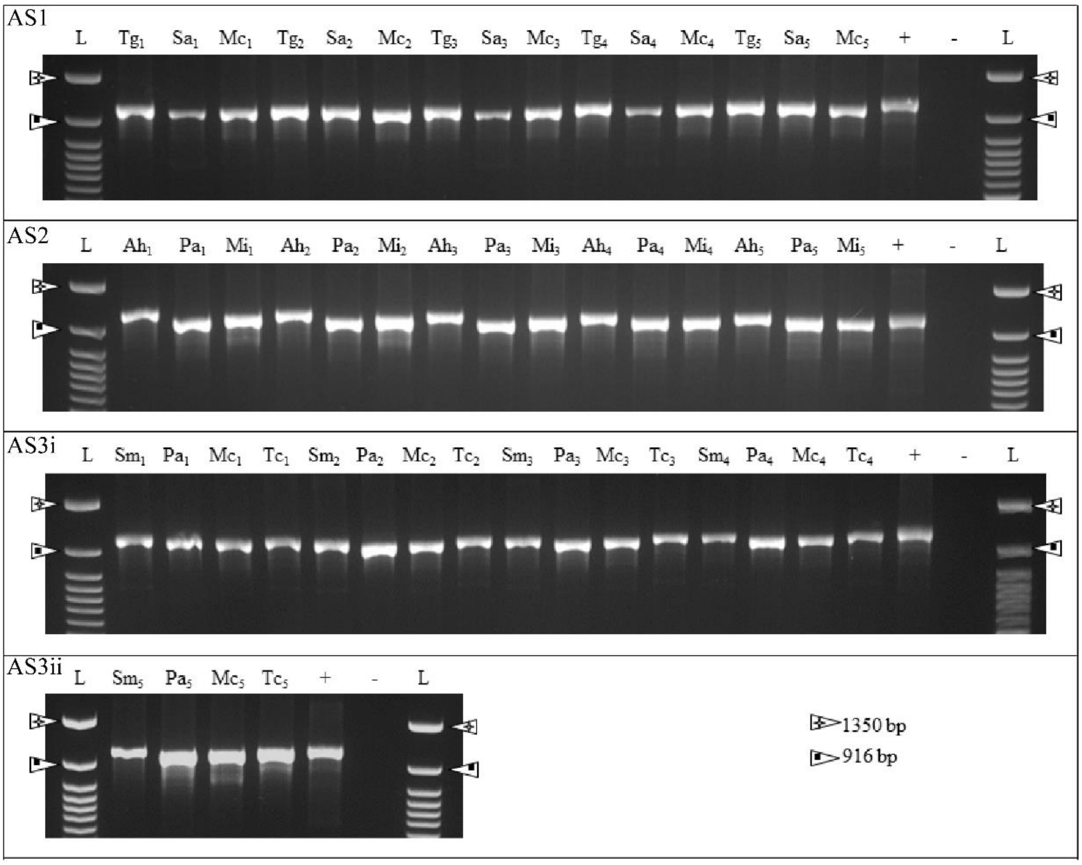

S12 Fig. ***atpB-rbcL*** **length polymorphism (size separate.d in 2.5 % agarose gel electrophoresis) obtained using template DNA extracted from immature leaves.** AS1 (Tg: *Tectona grandis*, Sa: *Samanea saman*, Mc: *Magnolia champaca*); AS2 (Ah: *Artocarpus heterophyllus*, Pa: *Persea americana*, Mi: *Mangifera indica*; AS3 (Sm: *Swietenia macrophylla*, Pa: *P. americana*, Mc: *M. champaca* and Tc: *Toona ciliata*). Five trees from each species (1→5 in subscript numbers) are shown. L: 50 bp ladder; +: rice DNA as template DNA for positive control; -: negative control (i.e. without template DNA). Two of the standard band sizes of the ladder are shown two types of symbolic arrowheads.

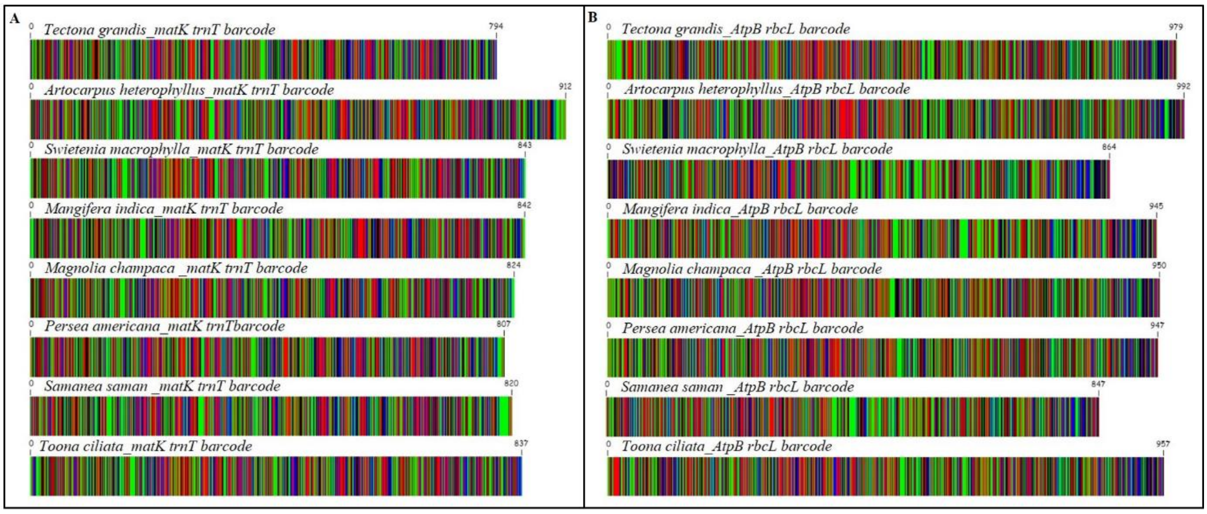

S13 Fig. DNA barcodes generated for three luxurious and five adulterant timber species. A: *matK-trnT*; B: *atpB-rbcL*.
